# Adapting Elder Mistreatment Screening for Home-Based Primary Care: The DETECT-RPC Project

**DOI:** 10.1093/geroni/igaf122.090

**Published:** 2025-12-31

**Authors:** Michael Cannell, Kristin Lees Haggerty, Jason Burnett, Melvin Livingston, Carolyn Pickering

**Affiliations:** TCU, Fort Worth, Texas, United States; Education Development Center, Waltham, Massachusetts, United States; UTHealth Houston, Houston, Texas, United States; Emory University, Atlanta, Georgia, United States; UTHealth Houston, Houston, Texas, United States

## Abstract

Elder mistreatment (EM) is underrecognized and underreported in home-based primary care (HBPC) settings, where clinicians often serve highly vulnerable older adults, including those with Alzheimer’s Disease and Related Dementias (ADRD). The Detection of Elder Mistreatment Through Emergency Care Technicians – Revised for Primary Care (DETECT-RPC) Project adapts a previously validated EMT-based EM screening tool for HBPC clinicians. Guided by the Theory of Planned Behavior (TPB) and the Abuse Intervention Model (AIM), DETECT-RPC seeks to overcome key barriers to EM screening, including clinician uncertainty about EM recognition, concerns about mandatory reporting, and lack of training. Through an intervention mapping process, we developed the DETECT-RPC screening tool to integrate into HBPC workflows and designed a training program to enhance clinician knowledge and confidence in identifying and responding to EM. We are currently conducting a cluster randomized controlled trial (RCT) to evaluate DETECT-RPC’s impact on screening and APS reporting behaviors among HBPC clinicians. This presentation will highlight findings from our formative research, the intervention development process, and early insights from clinician training and survey responses measuring attitudes and beliefs about EM reporting. Discussion will focus on implementation challenges, clinician concerns about APS involvement, and strategies for adapting EM screening tools to non-acute care settings. By addressing these barriers, DETECT-RPC aims to improve the identification and response to EM in home-based settings, providing a scalable model for EM screening beyond emergency and hospital-based care.

